# Assessment of Functional Outcomes of Open Carpal Tunnel Release Through the QuickDASH Questionnaire in Patients With Carpal Tunnel Syndrome: An Observational Retrospective Study

**DOI:** 10.7759/cureus.111002

**Published:** 2026-06-16

**Authors:** Rajiv K Bharti, Nikhilesh K Gaur, Anupama Singh, Anup Kumar, Ankur Bhatnagar

**Affiliations:** 1 Plastic Surgery and Burns, Sanjay Gandhi Postgraduate Institute of Medical Sciences, Lucknow, IND; 2 Biostatistics and Health Informatics, Sanjay Gandhi Postgraduate Institute of Medical Sciences, Lucknow, IND

**Keywords:** carpal tunnel release, carpal tunnel syndrome, entrapment neuropathy, median nerve decompression, quickdash

## Abstract

Purpose

Carpal tunnel syndrome (CTS) is the most common compressive neuropathy of peripheral nerves. The symptoms include numbness, paresthesia, night pain, and weakness of grip and/or hand function. Patient-reported outcome measures (PROMs) are now one of the most effective tools for the assessment of functional outcomes after CTS release. QuickDASH (a shortened, 11-item version of the Disabilities of the Arm, Shoulder and Hand (DASH) questionnaire) has evolved as one of the best questionnaire-based patient-reported outcome measure tools commonly used nowadays.

Materials and methods

We studied 35 patients from January 2023 to December 2025 at our institute. Diagnosis of CTS was based purely on clinical examinations and neurophysiological studies. The results were assessed and compared using QuickDASH questionnaire scores on two occasions: one preoperatively and another six months post-procedure.

Results

Patients were predominantly female (88.57%). Most patients were in the 41-60-year age group (51.43%). A total of 94.29% (n = 33) of individuals were right-hand dominant. Hand-specific disease laterality was 20 (57.14%) right hands and 15 (42.86%) left hands out of 35 hands. A total of 77.14% of patients showed a QuickDASH score below 25 at six months post-surgery, whereas 85.72% had scored above 50 during preoperative evaluation.

Conclusion

The QuickDASH questionnaire is a well-established and convenient tool to assess CTS outcomes. The study suggests that surgical decompression provides excellent improvement and results in all study subjects. However, even within the DASH scores, there is inter-item variability, with some points having better outcomes than others. This must be emphasized during preoperative patient counseling.

## Introduction

Carpal tunnel syndrome (CTS) is the most common upper limb compressive neuropathy and occurs due to entrapment of the median nerve at the wrist level [1]. Characteristic symptoms are numbness, tingling, and pain in the affected hand, especially during sleep, and difficulties in performing day-to-day work-related activities [2]. Sensory symptoms precede motor involvement [3]. The pathophysiology involves a combination of mechanical trauma, increased pressure, and ischemic injury to the median nerve within the carpal tunnel. A vicious cycle of local metabolic alterations, venous congestion, and ischemia is activated by repetitive mechanical forces. Release of inflammatory cytokines and attraction and activation of macrophages lead to demyelination, axonal degeneration, and, in the chronic setting, fibrosis of the nerve [4,5].

Individuals with systemic disorders such as hypothyroidism, diabetes, and rheumatoid arthritis have bilateral involvement, whereas most industrial workers have dominant hand involvement [6]. CTS is managed either by conservative means or by surgical interventions. Conservative treatment usually includes a combination of nonsteroidal anti-inflammatory drugs (NSAIDs), vitamins B6 and B12, local and/or oral corticosteroids, diuretics, ultrasound, low-dose laser, physiotherapy, and the use of hand splints [7,8]. Open release is widely considered the gold standard for the management of CTS [9,10]. Outcomes of carpal tunnel release are usually evaluated with patient-reported outcome measures (PROMs) [2]. An ideal evaluation tool has reproducibility, validity, internal consistency, and the ability to respond to clinical changes [11]. The QuickDASH (a shortened, 11-item version of the Disabilities of the Arm, Shoulder and Hand (DASH) questionnaire) scoring system appears to be an effective and practical method for the assessment of CTS [12].

The QuickDASH consists of 11 items and is scored from 0 (best) to 100 (worst). In this scoring system, at least 10 of the 11 items must be completed for score calculation. Each question is scored from 1 to 5, and the assigned values for all completed items are summed and averaged. This value is then transformed to a score of 0 to 100 by subtracting one and multiplying by 25 [13].

## Materials and methods

Aim of the study

This study aimed to evaluate patient-reported functional recovery following open carpal tunnel release using QuickDASH and to characterize item-level variability in postoperative functional improvement.

Study objectives

The primary objective of this study is to evaluate functional outcomes following open carpal tunnel release using the QuickDASH questionnaire by comparing preoperative and six-month postoperative recovery scores. The secondary objective is to investigate inter-item variability in postoperative recovery across individual QuickDASH functional domains.

Study design

This was a single-center, observational retrospective study conducted at a tertiary care hospital in North India from January 2023 to December 2025 and included 35 patients. The diagnosis was made through a combination of clinical examination, ultrasound, and nerve conduction studies, with clinical examination being the most important determinant.

In this study, the clinical signs and symptoms included Durkan compression test, Phalen’s test, reverse Phalen’s test, nocturnal paresthesia, tingling/numbness in the radial three and a half digits and thumb, and grip weakness. In nerve conduction studies, a median motor distal latency threshold of >4.2 milliseconds, a median sensory distal latency threshold of > 3.5 milliseconds, and a conduction velocity threshold of < 50 m/s across the wrist segment were considered positive parameters for CTS. In high-resolution ultrasound, a cross-sectional area of the median nerve > 12 mm² at the carpal tunnel inlet was considered positive for CTS. However, in patients with equivocal diagnostic tests and clinical presentations, clinical signs and symptoms were the overriding criteria for the diagnosis of CTS.

Surgical Technique

All surgeries were performed under median nerve block, loupe magnification, and tourniquet control. A single curvilinear incision measuring 3-4 cm at the base of the palm along the defined axis (radial aspect of the ring finger) started from the distal palmar crease to just proximal to Kaplan’s cardinal line and 2-3 mm ulnar to the thenar crease (to avoid injury to the palmar cutaneous branch). The palmaris fascia is identified just beneath the subcutaneous fat by its longitudinally running fibers. The dissection goes deeper to the palmar fascia to locate the transverse carpal ligament, which runs transversely. After proper identification and exposure of the transverse carpal ligament, a small incision was made in it to release the median nerve, and, after that, a precise complete decompression of the nerve was performed with sharp dissection under vision. Complete release of the transverse carpal ligament was ensured with the popping up of the fat pad at the distalmost part of the ligament. No dissection was performed beyond this fat pad to prevent undue injury to the superficial palmar arch. Proximally, approximately 2-2.5 cm of the antebrachial fascia at the distal forearm was also released through the same incision to complete the release. Decompression of the median nerve was ensured, and after achieving proper hemostasis, the wound was closed in a single layer with nonabsorbable sutures. Epineurolysis was not performed in any case. All patients were placed in a small wrist-forearm splint after dressing. Perioperatively, all patients received oral antibiotics for three days.

Postoperative Protocol

In the first week, splintage was continued, and hand elevation above heart level was advised to prevent postoperative edema. The first dressing was performed on the fifth postoperative day. In the second week, suture removal was performed on the 12th postoperative day under aseptic precautions. Wrist-forearm splintage was continued. Smiley ball exercises were advised to prevent stiffness. Patients were allowed to write and type for short durations (10-15 minutes), but heavy lifting or work was avoided. From the third week onward, gentle scar massage and progressive active and passive range-of-motion exercises were advised, and night splintage was continued for another two weeks. Complete return to normal activities was allowed six weeks post-surgery.

A self-administered QuickDASH questionnaire was completed by the patients themselves in either English or Hindi preoperatively and six months after open carpal tunnel release. The scores were compared to assess functional outcomes using the minimal clinically important difference (MCID) between preoperative and postoperative groups.

Inclusion and exclusion criteria

Adult patients with clinically and neurophysiologically proven carpal tunnel syndrome requiring surgical release were included in this study, whereas patients with recurrent CTS, bony disorders (such as arthritis), associated neurological disorders, pregnancy, missing questionnaire responses, or incomplete follow-up up to six months were excluded from the study.

Data collection

The data were collected from the HIS (Hospital Information System) and patients' files in the record room.

Statistical analysis

Statistical analyses were conducted using R version 4.5.1 (R Foundation for Statistical Computing, Vienna, Austria) and the ggstatsplot package version 0.13.3. The Wilcoxon signed-rank test was used to assess statistical significance. The P-value displayed in the graph assesses the effectiveness of carpal tunnel release based on the QuickDASH scores displayed through paired violin plots. Nonparametric tests (the two-group Mann-Whitney U test and the three-group Friedman rank sum test) were used to assess the statistical significance of changes in QuickDASH scores between two variables (disease laterality and age groups).

## Results

The present study consisted of 35 patients with a mean age of 45.37 years (SD, 12.26), with a minimum age of 20 years and a maximum age of 82 years. Of the 35 subjects, 16 patients had bilateral hand involvement, 14 had only right-hand involvement, and five had only left-hand involvement.

Table 1 shows the demographic features of the patients, wherein 31 (88.57%) individuals were female, and four (11.43%) were male. A total of 51.43% (n = 18) fell within the 41-60-year age group, whereas 40% fell within the 20-40-year age group. Only three patients (8.57%) were older than 60 years. A total of 94.29% (n = 33) of individuals were right-hand dominant, and 5.71% (n = 2) were left-hand dominant. Patients were divided into three groups based on the duration of disease symptoms before surgical release: less than six months, 6 to 18 months, and more than 18 months. The majority of patients in our study belonged to the 6- to 18-month group (48.57%, n = 17), followed by the less than six months group (34.29%, n = 12), whereas 17.14% (n = 6) were symptomatic for more than 18 months before surgical intervention.

**Table 1:** Demographic description

Variable	Numbers (n = 35)	Percentage (%)
Gender (Female / Male)	31 / 4	88.57 / 11.43
Hand dominance (Right / Left)	33 / 2	94.29 / 5.71
Age groups (years): 20–40 / 41–60 / ≥61	14 / 18 / 3	40 / 51.43 / 8.57
Duration of disease (months): <6 / 6–18 / >18	12 / 17 / 6	34.29 / 48.57 / 17.14
Laterality of disease (Bilateral / Right / Left)	16 / 14 / 5	45.71 / 40 / 14.29

**Figure 1:**
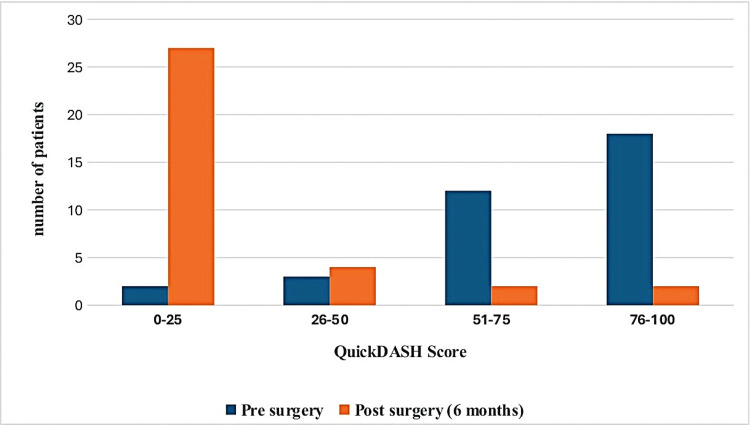
QuickDASH scores preoperatively and at six months postoperatively

Table 2 and Figure 1 show the changes in QuickDASH scores between the preoperative and six-month postoperative status of the patients. The score range was divided into four groups: 0-25, 26-50, 51-75, and 76-100. In the preoperative group, of the 35 patients, 51.43% (n = 18) fell within the 76-100 score range, and 34.29% (n = 12) fell within the 51-75 score range. Two patients had scores below 25 in the preoperative stage. In the six-month postoperative group, 77.14% (n = 27) showed scores below 25, 11.43% (n = 4) had scores between 26 and 50, and two (5.71%) individuals had scores above 75.

**Table 2:** Preoperative and six-month postoperative QuickDASH scores

Quick DASH scores (range)	Pre-surgery, n (%)	Post-surgery (six months), n (%)
0-25	2 (5.71)	27 (77.14)
26-50	3 (8.57)	4 (11.43)
51-75	12 (34.29)	2 (5.71)
76-100	18 (51.43)	2 (5.71)

**Figure 2:**
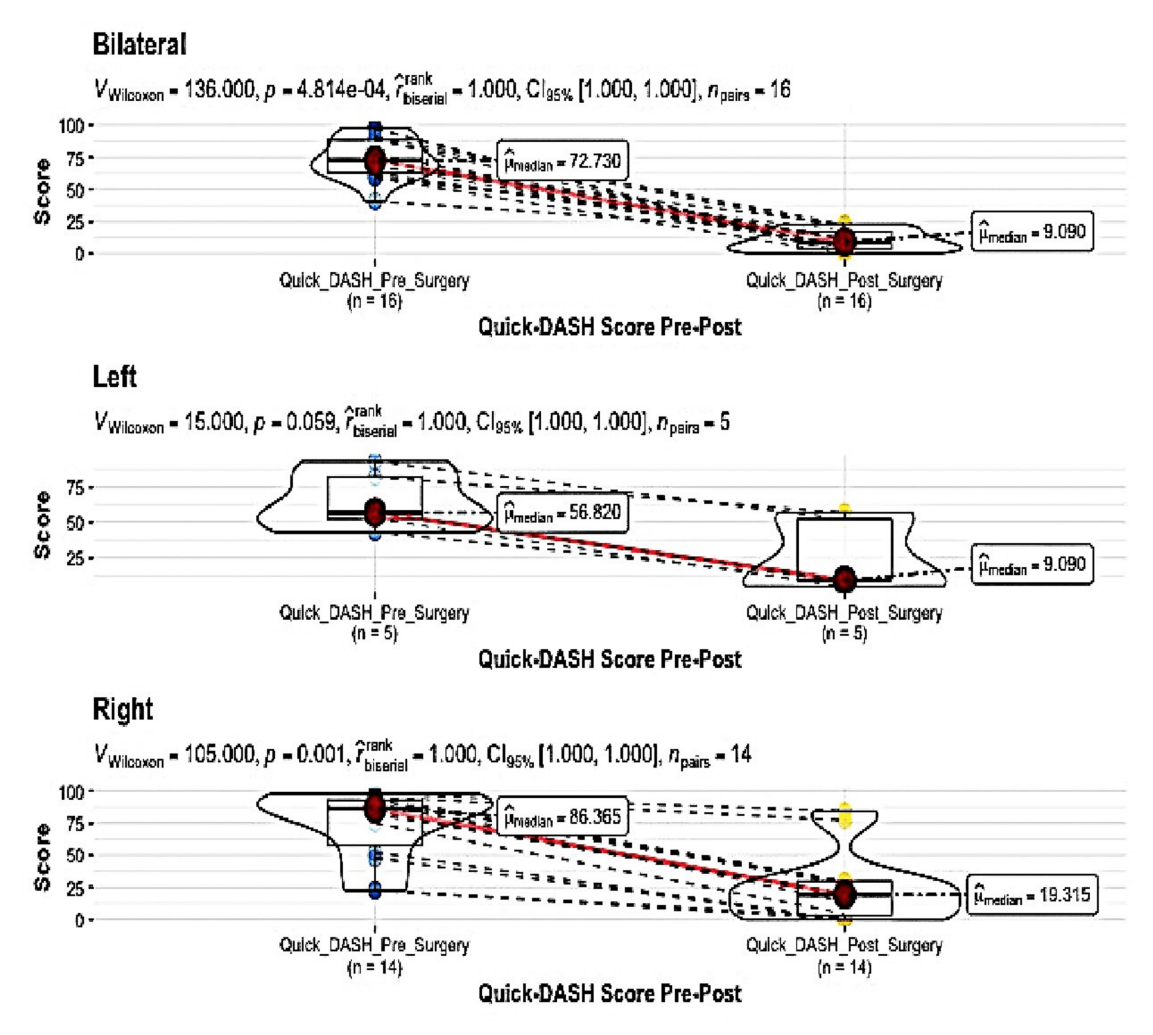
QuickDASH scores preoperatively and at six months postoperatively (by laterality)

Figure 2 shows laterality-wise changes in pre- and post-surgery QuickDASH scores. A total of 35 subjects were categorized by laterality (bilateral, left, and right). In bilateral cases, the mean score decreased significantly from 72.73 pre-surgery to 9.09 at six months post-surgery (p < 0.001). In right-sided cases, the mean score decreased from 86.365 pre-surgery to 19.315 at six months post-surgery (p = 0.001). In left-sided cases, the mean score decreased from 56.820 pre-surgery to 9.09 post-surgery (p = 0.059). This finding can be attributed to the very small sample size (only 5). Furthermore, two patients in this group presented very late (>18 months), as refractory changes due to median nerve compression had already been established.

**Figure 3:**
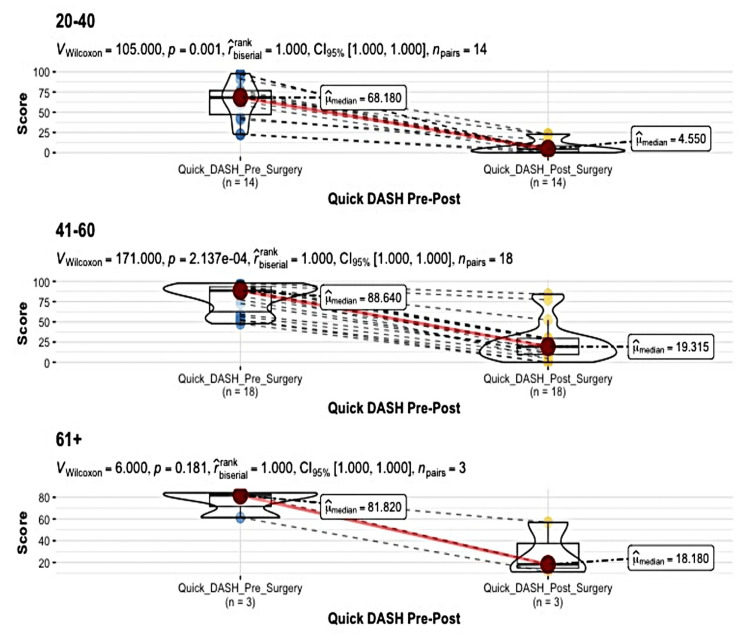
Preoperative and six-month postoperative QuickDASH scores by age group (years)

Figure 3 shows QuickDASH scores measured preoperatively and at six months post-surgery with respect to three age groups. The median score in the 20-40-year age group changed from 68.18 to 4.550, in the 41-60-year group from 88.64 to 19.31, and in the >60-year age group, it was from 81.82 to 18.18. The decrease in median scores across all age groups suggests that the changes were statistically significant.

**Figure 4:**
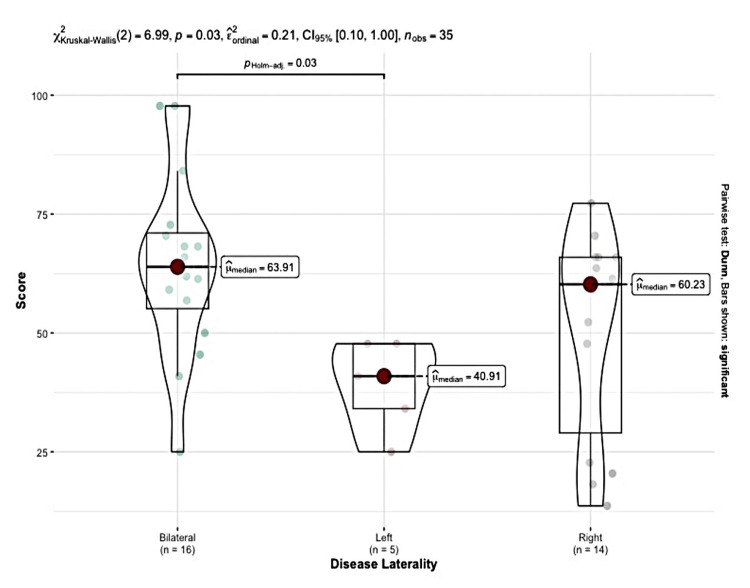
Distribution of changes in QuickDASH scores according to disease laterality

In Figure 4, the median changes according to disease laterality were -63.91, -60.23, and -40.91 for bilateral, right-sided, and left-sided cases, respectively. A p-value of 0.03 suggests a statistically significant correlation.

**Figure 5:**
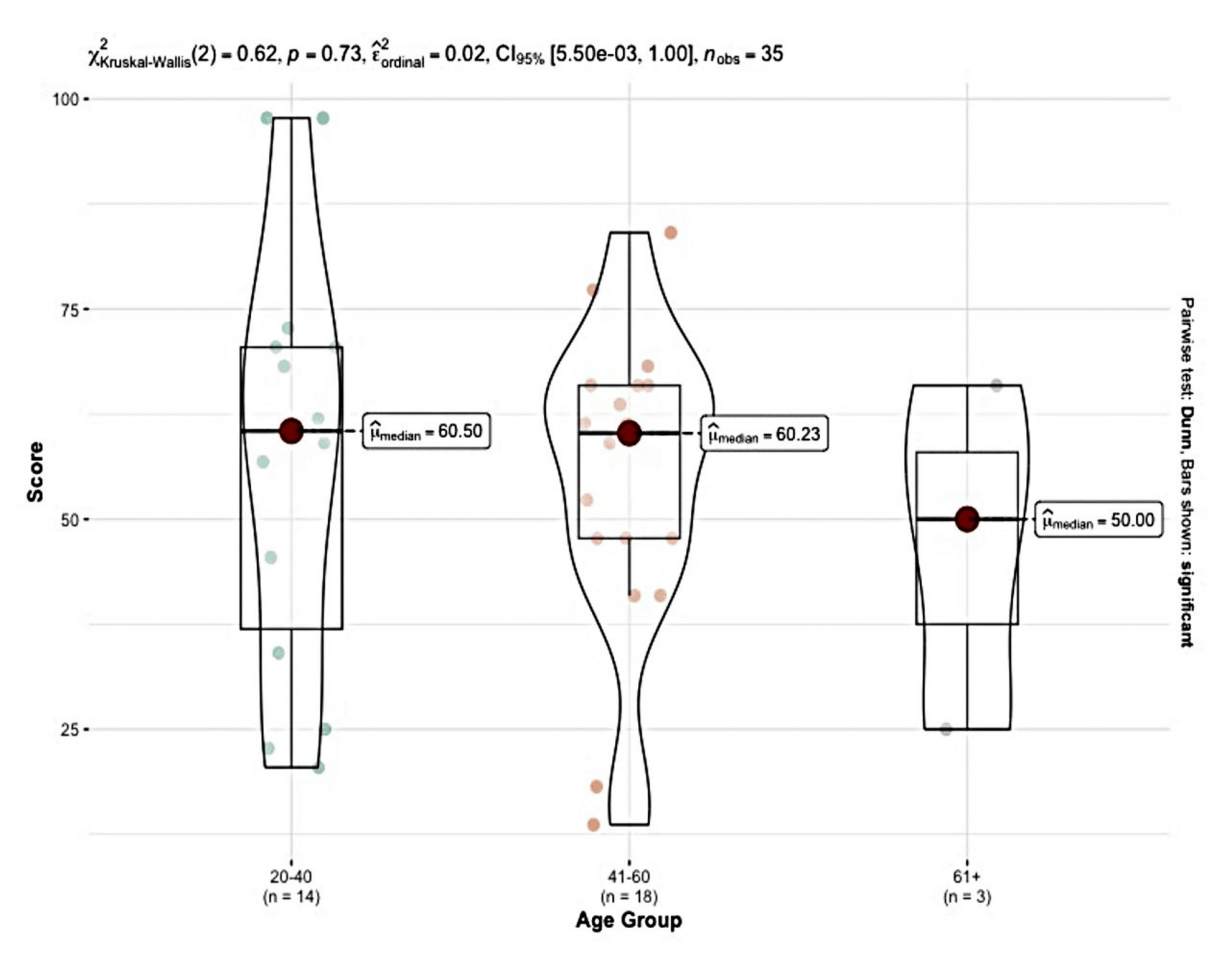
Distribution of changes in QuickDASH scores according to age group

Figure 5 shows the distribution of changes in QuickDASH scores across three age groups. The median score changes, indicated by red dots, were 60.50 for the 20-40-year age group, 60.23 for the 41-60-year age group, and 50.00 for the 61-plus age group. These findings indicate significant differences between the groups.

**Figure 6:**
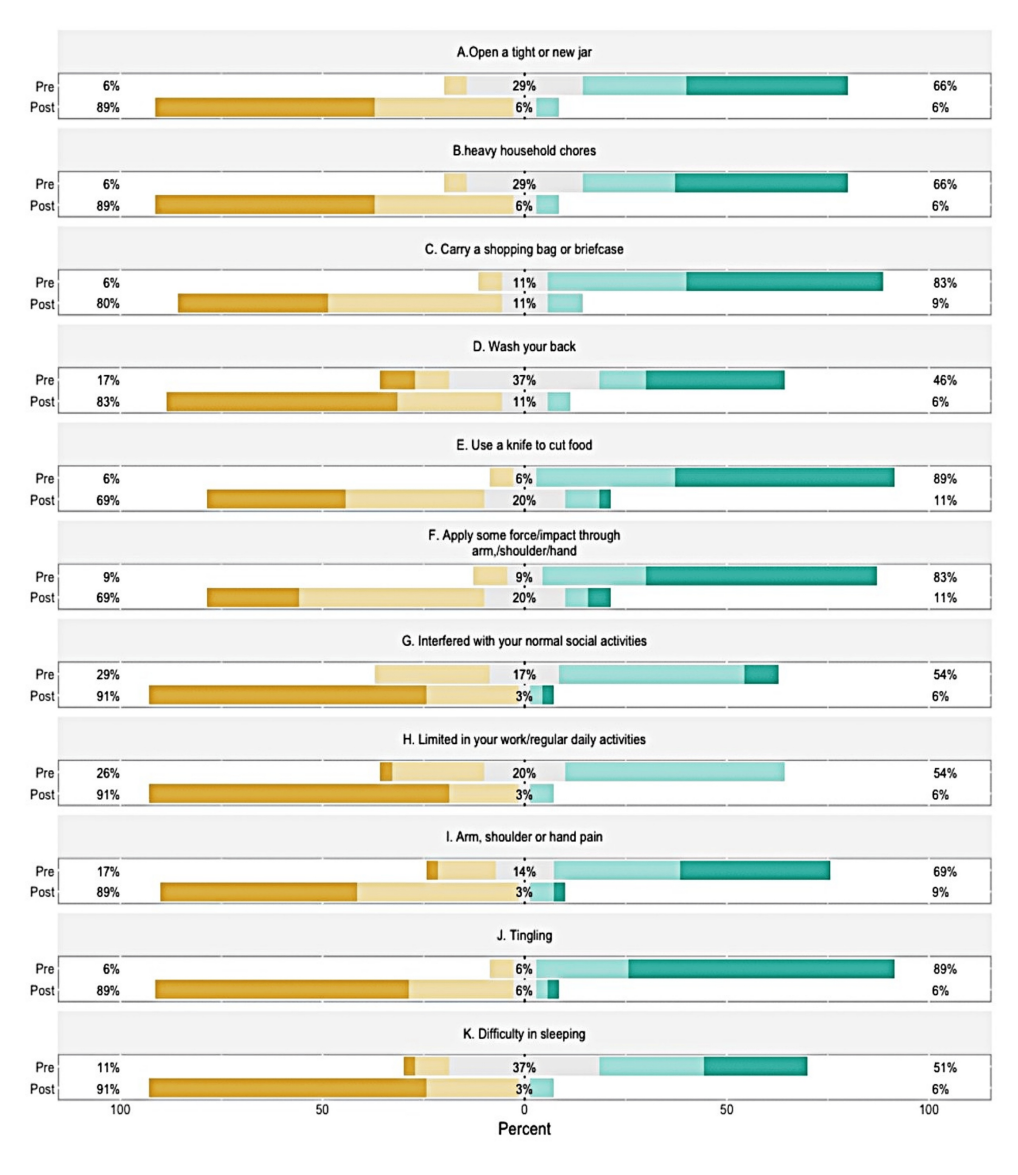
Inter-item variability assessment of QuickDASH scores in preoperative and six-month postoperative groups Stacked bar chart comparing preoperative and six-month postoperative data. The 11 items of the QuickDASH Likert-scale questionnaire are labeled A–K. Pre: Pre-surgery scores; Post: Six-month postoperative scores.

Figure 6 shows a simple representation of pre- and six-month post-surgery QuickDASH responses received from all patients through horizontal stacked bar charts, showing the percentage of responses for each activity. Each row represents a different activity. The data suggest excellent improvement in all responses, with a decrease in reported difficulty compared with the pre-surgery bars in >80% of patients. Only two activities, namely using a knife to cut food and recreational activities requiring some force, showed improvement in 69% of patients compared with the other activities.

## Discussion

Described by Paget in 1854, CTS is the most common type of compressive neuropathy and accounts for about 90% of entrapment neuropathies [1,12]. This syndrome is more common in middle-aged females [14]. de Krom et al. (1990) [15] and Nordstrom et al. (1998) [16] reported in their studies that the most common age of development for CTS is 45-60 years [12,14]. In our study, we found that the mean age of affected patients was 45.37 years with an SD of 12.26. According to Mondelli et al. (2002) [17] and Palmer et al. (2007) [18], the female preponderance was 72% and 83%, respectively, whereas in our study, the percentage of female patients was 88.57%.

Various studies have already been conducted to estimate the MCID for QuickDASH scoring between pre- and post-groups. According to Franchignoni et al. (2014) [19] and Kate Polson et al. (2010) [20], the MCID values were 15.91 and 19 points, respectively. Sorensen et al. (2013) [21] observed a baseline QuickDASH score of 50 and an MCID of 16.

In our study, we found that 85.71% (n = 30) of patients had preprocedural QuickDASH scores above 50, which improved significantly, dropping below 50 in 88.57% (n = 31) of patients. The overall mean QuickDASH scores preoperatively and at six months postoperatively were 72.92 and 17.92, respectively; hence, the overall mean difference was 55. Compared with the above-mentioned studies, the MCID in our study is much higher than the reported values, indicating excellent improvement in our cohort. A total of 27 (77.14%) of 35 patients showed excellent outcomes with a QuickDASH score below 25. Two patients did not show satisfactory improvement and were managed further as per the protocol for recurrent CTS cases. We also compared QuickDASH scores preoperatively and at six months postoperatively based on disease laterality and age groups, and both scenarios showed satisfactory improvement across all parameters.

Our study also assessed functional outcomes for each QuickDASH question, and the data indicated significant improvement in function and reduction in symptoms postoperatively across all tracked activities. However, activities requiring significant strength (knife cutting) and activities requiring multiple fine movements (recreational activities) showed lesser improvement in QuickDASH scores compared with routine hand activities.

The limitations of the study are that it is a single-center study with a small sample size. There is no control or comparison group, no postoperative electrophysiological assessment, and no long-term follow-up beyond six months. These may be addressed in future studies.

## Conclusions

This study suggests that surgical release gives overall excellent outcomes measured by QuickDASH for the management of CTS. However, inter-item variability was observed, as actions requiring significant force and good grip strength showed lesser improvement compared with other hand activities. Hence, patients requiring good grip strength during activities must be counseled preoperatively regarding the limited improvement of these symptoms.

## Figures and Tables

**Figure 7:**
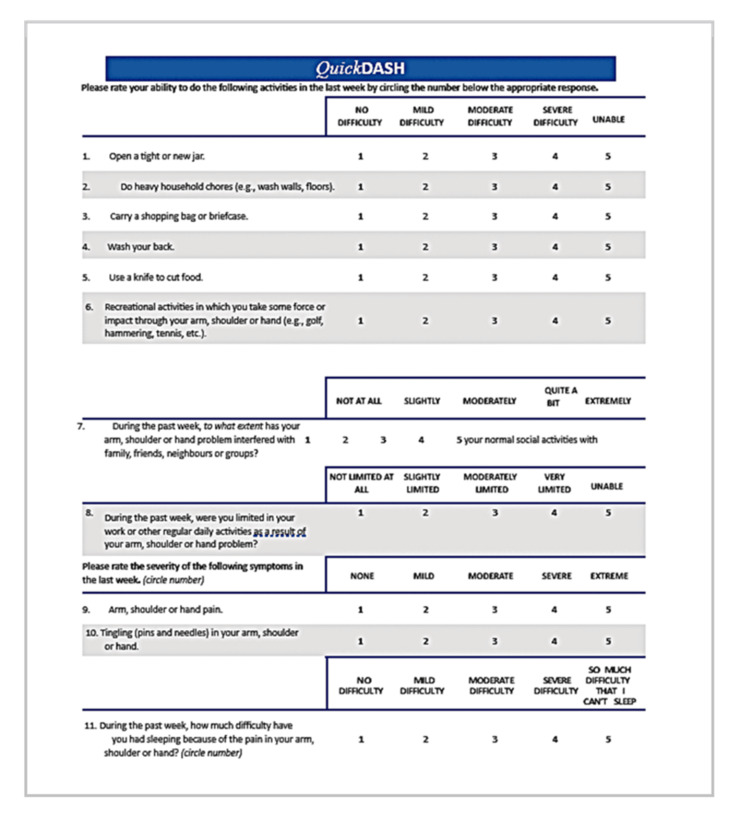
QuickDASH Questionnaire (English) Credit: Institute for Work & Health, Ontario, Canada (developers).

**Figure 8:**
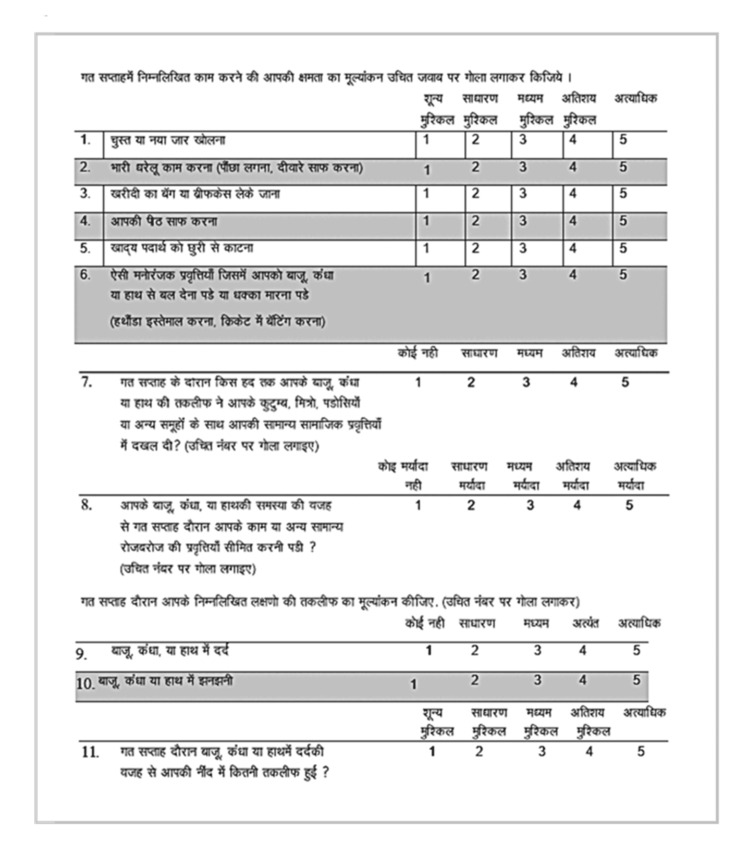
QuickDASH Questionnaire (Hindi) Credit: Institute for Work & Health, Ontario, Canada (developers).
